# Performance of Free‐Running Cardiac and Respiratory Motion‐Resolved Whole‐Heart 5D MRI in Congenital Heart Disease Patients Using a Gadolinium Enhanced Fast‐Interrupted Steady‐State Sequence

**DOI:** 10.1002/jmri.70402

**Published:** 2026-06-24

**Authors:** Christopher W. Roy, Eva Vogeli, Alissia Megalo, Fabienne Dirbach, Jessica A. M. Bastiaansen, Jérôme Yerly, Marco Mueller, David Rodrigues, Jean‐Baptiste Ledoux, Juerg Schwitter, Milan Prša, Estelle Tenisch, Tobias Rutz, Matthias Stuber

**Affiliations:** ^1^ Department of Diagnostic and Interventional Radiology Lausanne University Hospital (CHUV) and University of Lausanne (UNIL) Lausanne Switzerland; ^2^ Service of Cardiology Lausanne University Hospital (CHUV) and University of Lausanne (UNIL) Lausanne Switzerland; ^3^ Department of Diagnostic, Interventional and Pediatric Radiology (DIPR), Inselspital Bern University Hospital Switzerland; ^4^ Translational Imaging Center (TIC) Swiss Institute for Translational and Entrepreneurial Medicine Bern Switzerland; ^5^ Center for Biomedical Imaging (CIBM) Lausanne Switzerland; ^6^ Swiss Innovation Hub Siemens Healthineers International AG Lausanne Switzerland; ^7^ Cardiac MRI Center of the University Hospital Lausanne (CRMC) Lausanne University Hospital (CHUV) Lausanne Switzerland; ^8^ Interventional MRI Center of the University Hospital Lausanne Lausanne University Hospital (CHUV) Lausanne Switzerland; ^9^ Faculty of Biology and Medicine University of Lausanne (UNIL) Lausanne Switzerland; ^10^ Division of Pediatric Cardiology, Mother‐Woman‐Child Department Lausanne University Hospital (CHUV) and University of Lausanne (UNIL) Lausanne Switzerland

**Keywords:** cardiac function, congenital heart disease, coronary magnetic resonance angiography, free‐running, whole‐heart

## Abstract

**Background:**

The performance of 5D imaging in CHD has been previously demonstrated using ferumoxytol rather than gadolinium.

**Purpose:**

To evaluate the performance of gadolinium enhanced 5D MRI relative to 2D and 3D MRI in CHD patients.

**Study Type:**

Retrospective.

**Subjects:**

45 consecutive CHD patients referred for clinically indicated MRI who underwent 2D, 3D, and 5D MRI.

**Field Strength/Sequence:**

2D bSSFP CINE, 3D bSSFP, and 5D FISS at 1.5 T.

**Assessment:**

Scan time (2D, 3D, and 5D), left and right ventricular (LV, RV) end diastolic and end systolic volumes (LVEDV, RVEDV, LVESV, and RVESV, respectively), ejection fraction (LVEF, RVEF) for 2D versus 5D, and image quality on a 4‐point Likert scale (3D vs. 5D).

**Statistical Tests:**

Paired comparisons were made using a paired *t*‐test or Wilcoxon signed rank test. Agreement between measurements was evaluated with Bland–Altman analysis including the bias and 95% limits of agreement.

**Results:**

Scan time for 5D (6:18 [0]) was significantly shorter than 2D (7:16 [4:39]) and 3D (6:55 [1:30]) MRI. 5D measurements were strongly correlated with 2D measurements of LVEDV (*R* ^2^ = 0.94), LVESV (*R* ^2^ = 0.83), LVEF (*R* ^2^ = 0.51), (*R* ^2^ = 0.85), RVESV (*R* ^2^ = 0.80), and RVEF (*R* ^2^ = 0.66) but with significant biases. Sharpness of the coronary arteries were significantly higher for 3D (RCA: 36.8% ± 10.2%, LAD: 40.5% ± 10.5%) than 5D (RCA: 24.3% ± 7.5%, LAD: 31.7% ± 8.7%), but longer vessel segments were visible using 5D (LAD: 6.9 ± 2.5) than 3D (LAD: 5.3 ± 2.9 mm) acquisitions. The grades for 5D (R1: 3 [1], R2: 2.5 [0.5], R3: 3 [0.6]) were significantly higher than 3D (R1: 3 [1], R2: 2.5 [1], R3: 2 [1]).

**Data Conclusion:**

Gadolinium‐enhanced 5D MRI may provide an efficient and simplified acquisition for CHD patients, with multiple quantitative parameters from 5D being not significantly different from those from reference standard 2D and 3D MRI.

**Evidence Level:**

4

**Stage of Technical Efficacy:**

1

## Plain Language Summary

A highly efficient and simplified method for dynamic imaging of the whole heart called five‐dimensional (5D) FISS is presented. This approach uses standard gadolinium contrast rather than previously demonstrated imaging that used off‐label ferumoxytol providing a method that can be more widely disseminated. Comparisons between 5D FISS with reference standard two and three‐dimensional imaging were performed in 45 patients with congenital heart disease. 5D FISS was not significantly different from reference standards across a number of relevant clinical parameters. These findings suggest this approach could simplify and improve cardiac imaging for patients with congenital heart disease.

## 1 Introduction

Two‐dimensional (2D) CINE imaging, and electrocardiogram (ECG) triggered respiratory motion compensated three‐dimensional (3D) whole heart imaging are important for assessing ventricular function and cardiac morphology, respectively in clinical cardiac MRI of congenital heart disease (CHD) patients [1, 2]. Despite the success of these approaches, a high degree of expertise and patient collaboration is required due to the need for precise slice prescriptions (2D), identifying periods of quiescent cardiac motion (3D), breath‐holding (2D), which is not tolerated by all patients, navigator placement (3D), and the use of sedation or general anesthesia, especially in children [3]. The advent of dynamic 3D cardiac MRI through 4D (cardiac motion resolved) or 5D (cardiac and respiratory motion resolved) imaging attempts to simplify exams by capturing the entire dynamic 3D cardiac anatomy without the need for prospective scan planning, ECG gating or breath‐holding [4] in a motion‐tolerant manner to reduce the need for sedation and anesthesia [5]. In CHD patients, free‐running 4D and 5D imaging has been shown to provide excellent delineation of the cardiac anatomy [5] and assessment of cardiac function [6] within single time‐efficient sequences. However, unlike reference standard 2D CINE imaging, which benefits from improved blood to myocardium contrast due to unsaturated blood flowing into each slice, volumetric acquisitions often suffer from poorer native contrast due to the saturation of magnetization within the entire 3D field‐of‐view [7]. Additionally, the presence of both pericardial and subcutaneous fat can lead to obscured visualization of the coronary arteries as well as accentuated ghosting or streaking artifact from hyperintense fat signal, creating the need for fat signal suppression strategies [8]. As a result of these challenges, 4D [9, 10, 11, 12] and 5D [5] imaging techniques for CHD have included the use of an iron‐based (ferumoxytol) contrast agent to achieve blood‐pool signal that is sufficiently high to delineate vessels even in the presence of pericardial fat. Ferumoxytol enhanced imaging has been shown to provide excellent blood‐myocardium contrast and high enough signal‐to‐noise ratio to achieve the spatial and temporal resolution required to evaluate the complex dynamic cardiac anatomy of CHD patients within a clinically acceptable scan time [5, 9, 12, 13]. However, ferumoxytol is an off‐label contrast agent [14] and there has therefore been limited dissemination of these 4D and 5D techniques. Conversely, gadolinium (Gd) enhanced imaging is commonly used in clinical imaging of CHD patients as well as in general for cardiac MRI [2, 15].

To build on the promise of 4D and 5D methods and provide a simplified solution for imaging CHD patients that can be more widely disseminated, this study proposed using a free‐running Gd enhanced fast‐interrupted steady‐state (FISS) sequence [16, 17, 18] to acquire cardiac and respiratory motion‐resolved 5D images in CHD patients. Given the existing widespread use of Gd as a contrast agent in CHD patients, and the inherent fat signal suppression properties of this FISS sequence, the hypothesis of this study was that Gd‐enhanced FISS can simultaneously assess cardiac dynamics and whole‐heart morphology with equivalent performance to reference standards.

Thus, the aim of this study was to compare the proposed Gd‐enhanced 5D FISS sequence to reference standard 2D CINE imaging and to an ECG‐triggered self‐navigated 3D whole heart sequence [19, 20, 21, 22] in a cohort of CHD patients using multiple quantitative metrics.

## 2 Methods

### 2.1 Patient Population and MR Imaging Acquisition

Patients with CHD (See Supporting Information) were scanned as part of a larger ongoing study approved by the local research ethics committee. Each patient provided informed written consent allowing for the addition of research MRI sequences to the clinical protocol. Forty‐five consecutive patients (34.9 ± 14.5 years of age, 21 female) were retrospectively included in the current study based on the criteria that they underwent all three of 2D CINE imaging, 3D whole‐heart imaging, and 5D imaging described as follows. 2D breath‐held bSSFP CINE imaging was performed prior to clinically indicated injection of 0.2 mmol/kg body weight of Gadobutrol (Gadovist, Bayer Schweiz AG, Switzerland). Self‐navigated ECG‐triggered 3D radial bSSFP whole‐heart imaging [19, 20, 21, 22] with a water‐excitation pulse to minimize signal from fat, and free‐running 5D FISS whole‐heart imaging [17, 23] with native fat suppression, were performed after contrast injection. For simplicity, these sequences are referred to as 2D, 3D, and 5D hereafter. All 3D acquisitions were acquired during a prospectively chosen mid‐diastolic resting phase based on the ECG. All scans were performed on a 1.5 T clinical MR scanner (MAGNETOM Sola, Siemens Healthineers AG, Forchheim, Germany) with an 18‐channel surface coil, 32‐channel spine coil and the scan parameters listed in Table 1. The acquisition time and average patient heart rate for 2D, 3D, and 5D sequences was recorded for subsequent comparison. For 2D acquisitions, this included the time to acquire each slice and any pauses between breath‐holds. The order of acquisitions was always 2D followed by 3D, and then 5D due to the need for 2D and 3D imaging in the clinical protocol and the addition of 5D imaging as a research sequence. The acquisition time post injection of Gadobutrol was therefore recorded for both 3D and 5D imaging to evaluate any bias with respect to time post injection.

**TABLE 1:** Sequence parameters.

	2D	3D	5D
Excitation	Selective	Selective	Non‐selective
Acquired in‐plane resolution (mm)	1.17–1.56	1.14–1.25	1.25
Slice thickness (mm)	8	1.14–1.25	1.25
Slab thickness (mm)	—	220–240	—
Temporal resolution (ms)	19.2–45.9	—	25–49
Triggered acquisition window (ms)	—	76–133	—
TR (ms)	2.92–3.18	3.02–3.85	3.04
TE (ms)	1.28–1.39	1.52–1.87	1.52
RF excitation angle (°)	63–80	97	59–82
Bandwidth (Hz/Pixel)	930–977	1132	980
Spokes per radial interleave	—	25–35	24
Number of radial interleaves	—	351–479	3419
Total number of radial spokes	—	11,460–12,285	82,056

### 2.2 MR Image Reconstruction

2D and 3D images were generated by the scanner's online reconstruction. The 3D reconstructions included translational motion correction of respiration along the superior–inferior direction [19]. The free‐running acquisitions were reconstructed as cardiac and respiratory‐motion resolved 5D images using a previously described fully self‐gated compressed sensing approach [24, 25, 26] coded in MATLAB (MathWorks, Natick, Massachusetts) and performed offline on a workstation. All compressed sensing reconstructions were performed using an alternating direction method of multipliers (ADMM) algorithm with the augmented Lagrangian fixed to 0.06 and a total of 8 ADMM iterations. Regularization weights along the spatial, cardiac, and respiratory dimensions were empirically set to 0.001, 0.1, and 0.1, respectively. The workstation was equipped with 2 Intel Xeon CPUs (Intel, Santa Clara, California), 512 GB of RAM, and a NVIDIA Tesla GPU (Nvidia, Santa Clara, California). The total reconstruction time was approximately 8 h per 5D dataset.

### 2.3 MR Imaging Analysis

The end‐diastolic, end‐systolic, and mid‐diastolic cardiac phases were manually identified for each 2D and 5D dataset (3D data was prospectively acquired during mid‐diastole only) by a reviewer with 15 years' experience in cardiac MRI (CWR). Additionally, the end‐expiratory respiratory state was automatically identified [27] for 5D data and chosen for all qualitative and quantitative assessments. For comparisons of the three sequences in specific cardiac views (i.e., short‐axis), a script written in MATLAB was used to automatically reformat 3D and 5D data into the desired view based on the known coordinates of the 3D/5D data and the prospectively prescribed 2D orientations. Volumetric 3D renderings of the reconstructed 5D data were created in MATLAB to visualize the heart during end‐diastole and end‐systole.

Quantitative comparisons of all three sequences were performed by measuring the blood‐myocardium contrast, blood‐myocardium interface sharpness, and blood‐fat contrast as follows. For a given dataset, a region of interest (ROI) was manually drawn (CWR) on mid‐ventricular short‐axis views for 2D, 3D, and 5D images in the left ventricular blood‐pool and in the myocardium. The average signal intensity ratio between these two ROIs was defined as the blood‐myocardium contrast. Similarly, 10 line segments were manually (CWR) placed perpendicular to a semicircular portion of the left ventricular blood‐myocardium boundary and the average slope of sigmoid functions fit to the signal along these line segments was defined as the blood‐myocardium interface sharpness [28]. The slope calculation took into account the differences in acquired spatial resolution. Finally, a ROI was manually drawn (CWR) in the left ventricular blood‐pool and in the fat of the chest wall. The average signal ratio between these two ROIs was defined as the blood‐fat contrast. For 3D and 5D data the blood‐myocardium contrast and interface sharpness were additionally assessed as a function of time post injection of Gd to evaluate the impact of contrast agent on these measurements.

Quantitative comparison of the dynamic cardiac cycle as assessed by 2D and 5D imaging was performed by measuring left and right ventricular volumes during end‐diastole (LVEDV, RVEDV), end‐systole (LVESV, RVESV), and the ejection fraction (LVEF, RVEF). For all 2D images and reformatted 5D images, segmentation of the left and right ventricle was performed on the multi‐slice 2D CINE data by two medical students (EV, AM, each with 1 year experience) supervised by a cardiologist (TR with 15 years' experience) using Circle CVI 42 (Circle Cardiovascular Imaging, Calgary, Canada).

Multiplanar reformats of the right (RCA) and left anterior descending (LAD) coronary arteries were performed by a reviewer (CWR with 15 years' experience) and measurements of the vessel sharpness (percent change relative to centerline measurement) and vessel length were recorded for all prospectively acquired 3D images and manually selected mid‐diastolic resting phase images from the 5D data. All reformats and measurements were performed using SoapBubble [29].

Finally, all 3D and 5D images were evaluated by three reviewers (ET radiologist with 12 years' experience, FD cardiologist with 2 years' experience, TR cardiologist with 15 years' experience) using a 5‐point Likert scale: non‐diagnostic (0), marked blurring limited diagnostic value (1), moderate blurring diagnostic value (2), mild blurring good diagnostic value (3), excellent diagnostic quality (4).

For all qualitative and quantitative measurements, the order of images was randomized, and the observers were blinded to the patient's information.

### 2.4 Statistical Analysis

Data were tested for normality with the Shapiro–Wilk test and subsequent values are reported as the mean ± standard deviation or median [interquartile range]. Paired comparisons were made using a paired *t*‐test for normal data and a Wilcoxon signed rank test otherwise. Linear regression, intraclass correlation coefficient (ICC), and standard error measurement (SEM) were calculated to compare volumetric measurements between sequences. Correlation was defined as negligible (*r* < 0.10), weak (0.10–0.39), moderate (0.40–0.69), strong (0.70–0.89), and very strong (0.90–1.00) [30]. ICC was classified as poor (ICC < 0.4), fair (0.40–0.59), good (0.60–0.74), and excellent (0.75–1.00) [31]. Agreement between measurements was evaluated with Bland–Altman analysis including the bias and 95% limits of agreement. A *p* value lower than 0.05 was considered statistically significant after Bonferroni correction for multiple comparisons.

## 3 Results

**FIGURE 1:**
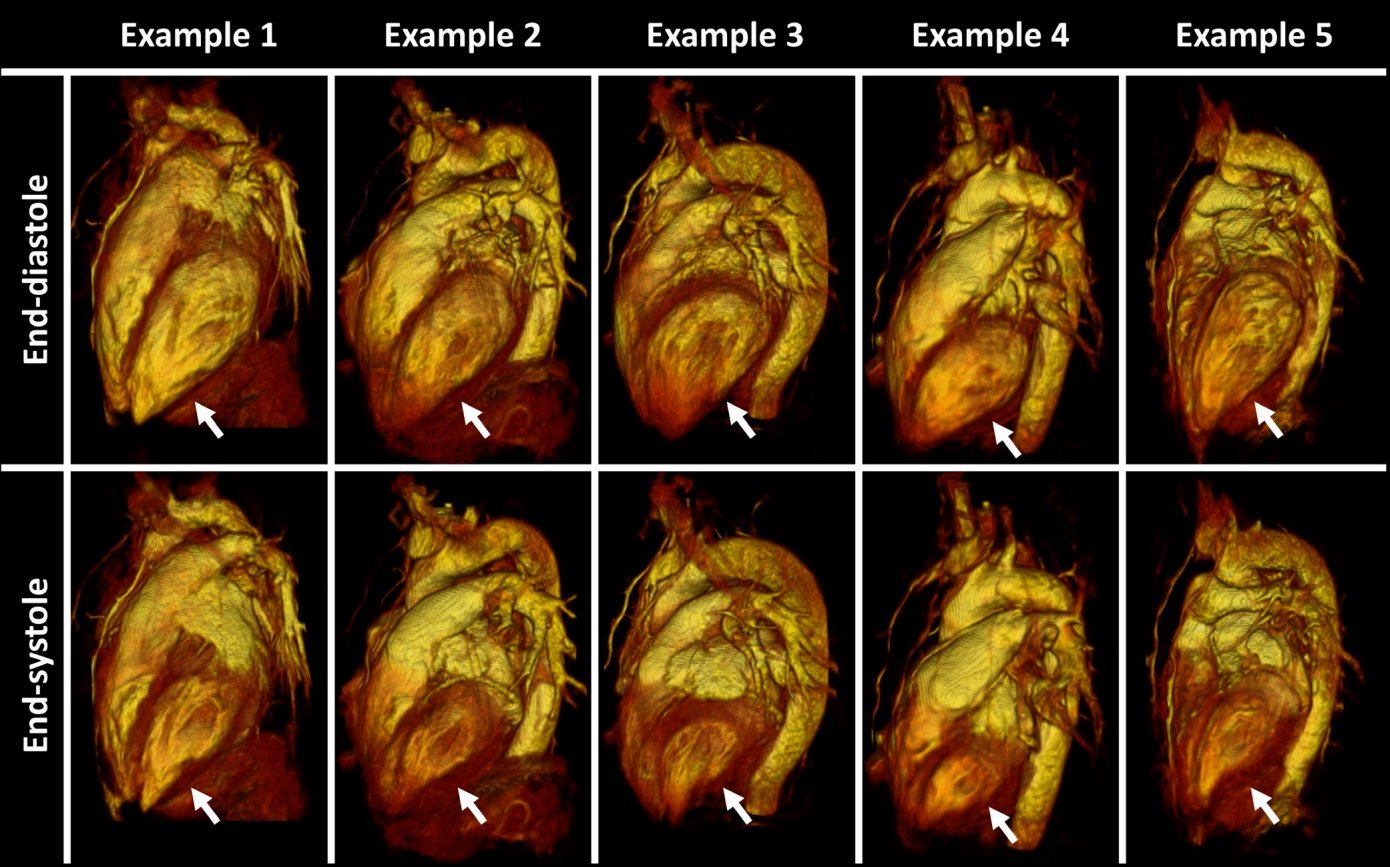
Free‐running 5D FISS volumetric 3D renderings of the heart at end‐diastole and end‐systole enabling clear depiction of left ventricular contraction (white arrows) in five example subjects.

All free‐running FISS acquisitions were successfully reconstructed as cardiac and respiratory motion‐resolved 5D images providing visualization of the entire 3D cardiac anatomy throughout the cardiac cycle (Figure 1). Free‐running 5D had a fixed scan time of 6:18 min which was significantly shorter than 2D (7:16 [4:39]) and 3D scan times (6:55 [1:30]). There were no significant differences in patient heart rates between 2D (65 ± 10) and 3D (66 ± 8, *p* = 0.16), 2D and 5D (67 ± 11, *p* = 0.10) or 3D and 5D (*p* = 0.42).

**FIGURE 2:**
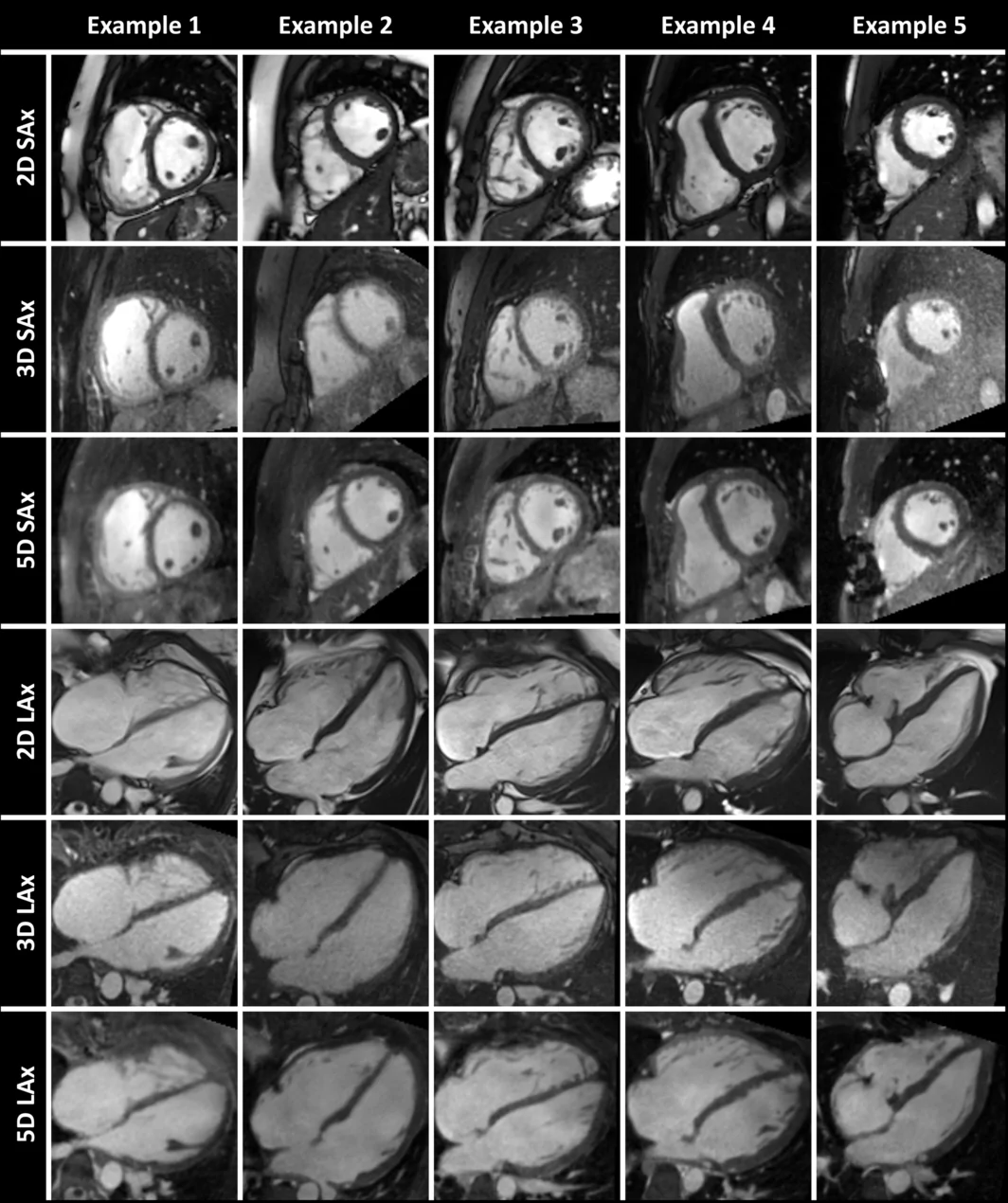
Double‐oblique reformats of 2D, 3D, and 5D images showing short‐axis (first, second, and third rows) and long‐axis (fourth, fifth, and sixth rows) views for five example subjects.

**FIGURE 3:**
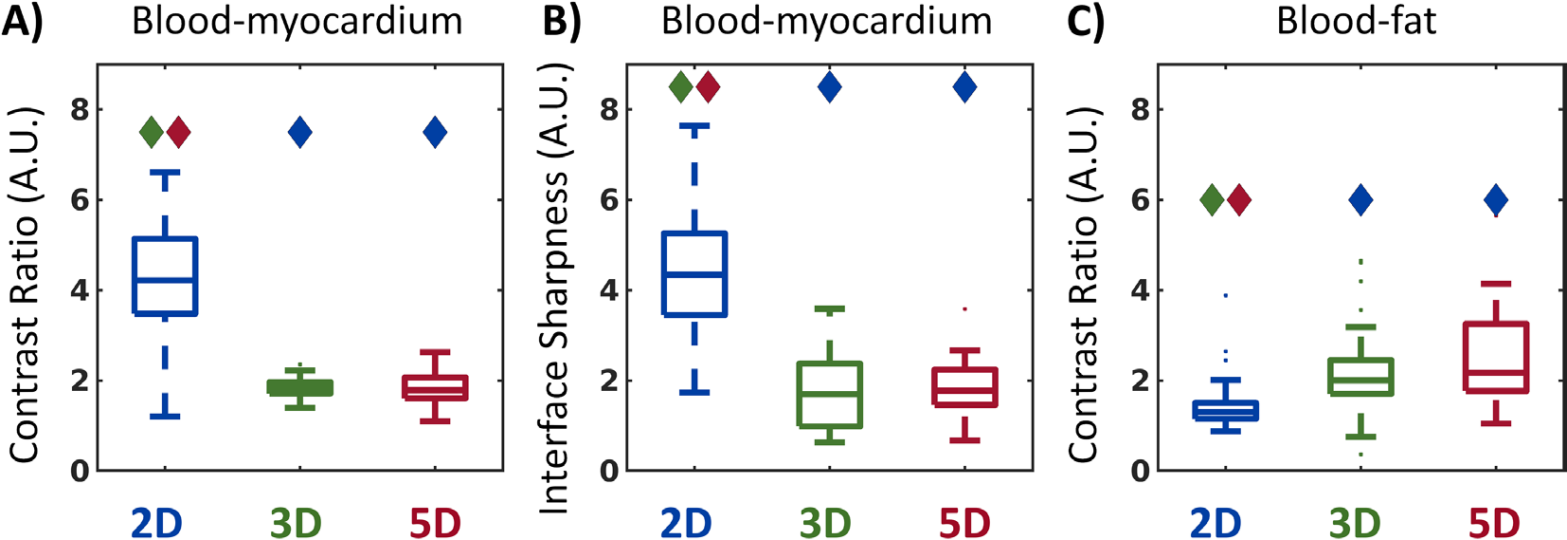
Quantitative comparison of 2D, 3D, and 5D images. Boxplots for blood‐myocardium contrast (A), blood‐myocardium interface sharpness (B), and blood‐fat contrast (C) show the total range, with the median indicated by the central mark and the 25th and 75th percentiles by the bottom and top box edges, respectively. Colored diamonds denote significant differences (*p* < 0.05) between the three imaging methods.

Retrospective short‐axis reformats of 3D and 5D data were successfully performed for subsequence comparison to prospectively acquired 2D short‐axis images (Figure 2). The blood‐myocardium contrast (Figure 3a) for 2D (4.22 [1.70]) was significantly higher than for 3D (1.83 [0.34]) and 5D (1.77 [0.45]); however, there was no significant difference in the blood‐myocardium contrast between 3D and 5D (*p* = 0.85). Sharpness measured along the blood‐myocardium interface (Figure 3b) for 2D (4.13 ± 1.58) was also significantly higher than for 3D (1.67 ± 0.88) and 5D (1.73 ± 0.70), but the difference in interface sharpness measured between 3D and 5D (*p* = 0.66) was not significant. The blood‐fat contrast (Figure 3c) for 2D (1.28 [0.38]) was significantly lower than for 3D (1.95 [1.00]) and 5D (2.20 [1.56]), but again there was no significant difference in the blood‐fat contrast between 3D and 5D (*p* = 0.07).

**FIGURE 4:**
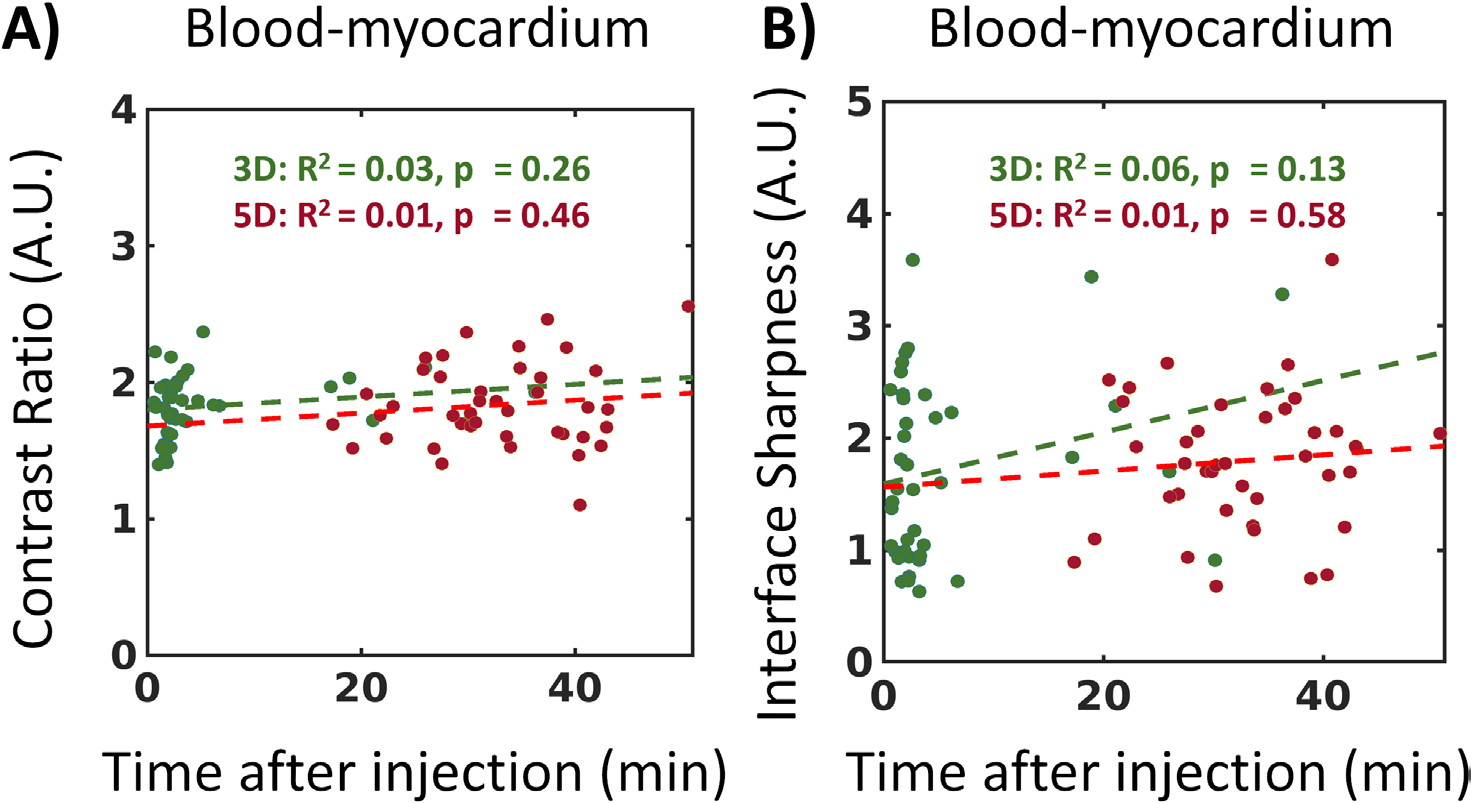
Quantitative evaluation of blood‐myocardium contrast (A) and interface sharpness (B) as a function of acquisition time post gadolinium injection for 3D and 5D.

A significantly larger range of acquisition times post injection of Gd for 5D (32:37 [11:26]) was recorded than for 3D (2:14 [2:18]). Neither the blood‐myocardium contrast (Figure 4a) for 3D (*R* ^2^ = 0.03, *p* = 0.26) and 5D (*R* ^2^ = 0.01, *p* = 0.46) nor the interface sharpness (Figure 4b) for 3D (*R* ^2^ = 0.06, *p* = 0.13) and 5D (*R* ^2^ = 0.01, *p* = 0.58) showed significant change as a function of time post injection of Gd.

**FIGURE 5:**
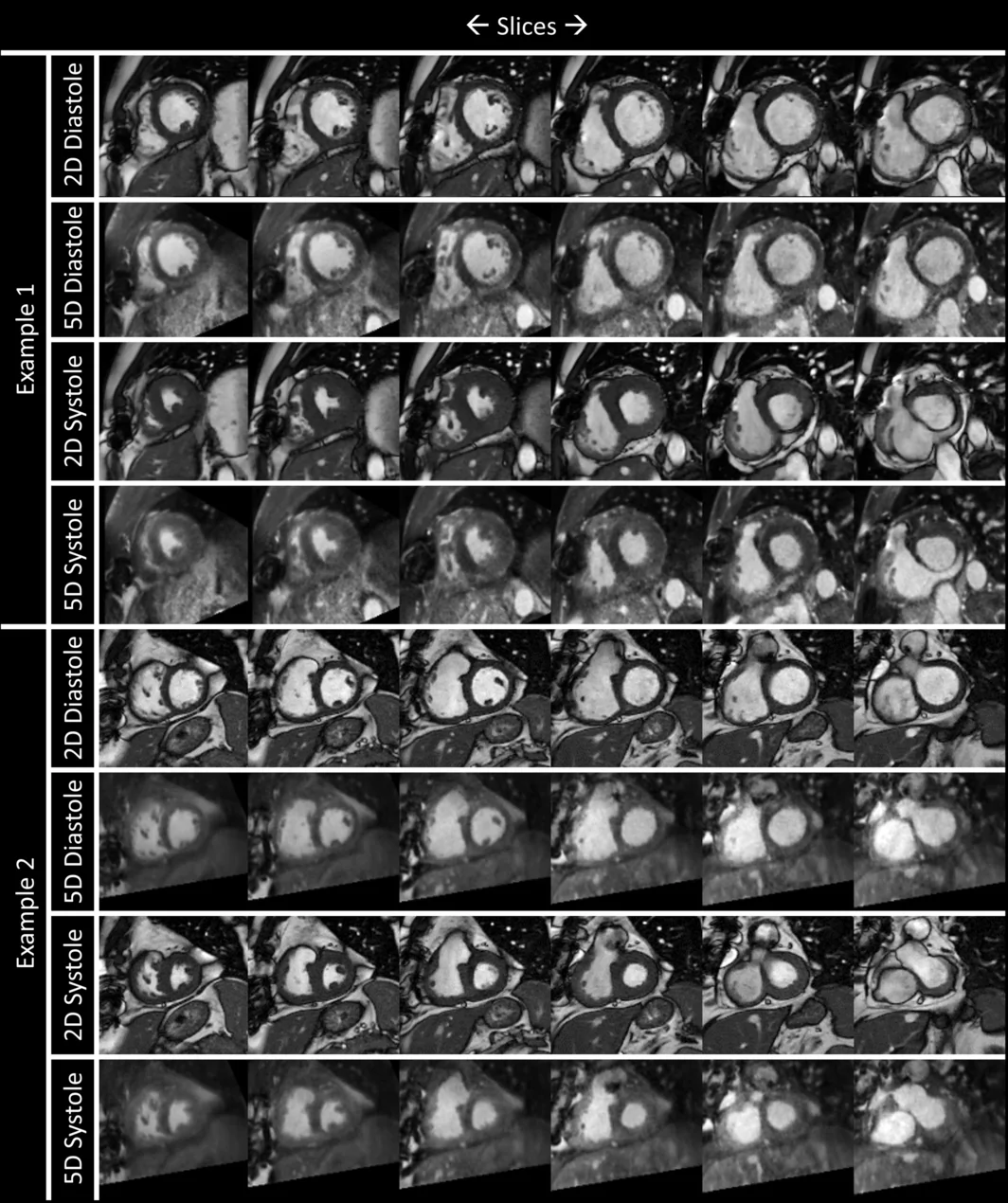
2D CINE images in short‐axis orientations and reformatted 5D images: Matching slices are shown at end‐diastole and end‐systole for two example subjects.

**FIGURE 6:**
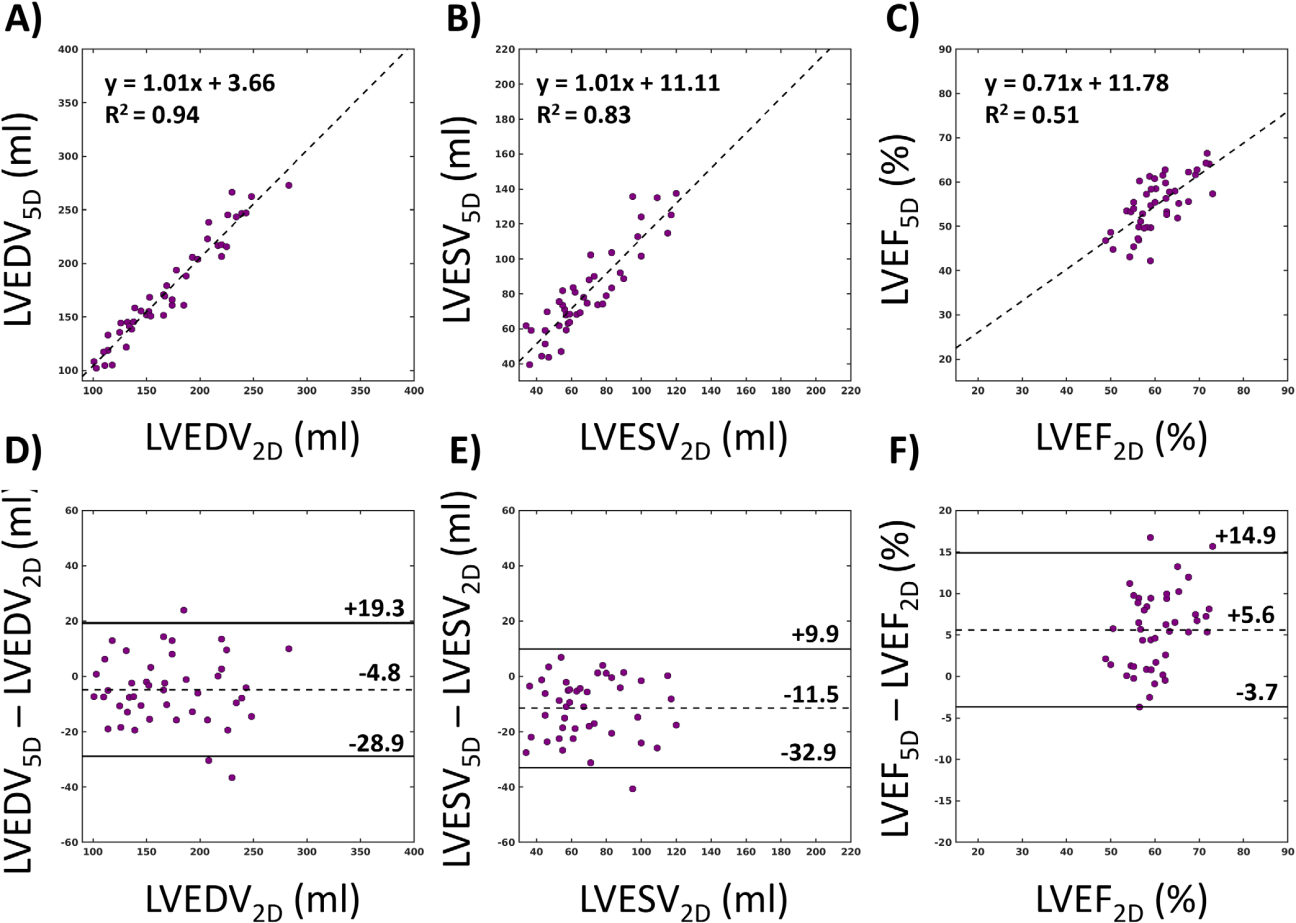
Quantitative evaluation of left ventricular volumetry derived from 2D and 5D imaging data. End‐diastolic volume (A&D), end‐systolic volume (B&E), and ejection fraction (C&F) are assessed by correlation plots (A–C) and Bland–Altman analyses (D–F).

End‐diastole and end‐systole were successfully reformatted as short‐axis multi‐slice images for comparison to 2D images across multiple slice positions (Figure 5). For the left ventricle, strong to very strong correlation (Figure 6) was found between 2D and 5D measurements of LVEDV (*R* ^2^ = 0.94), LVESV (*R* ^2^ = 0.83), and LVEF (*R* ^2^ = 0.51) across the range of measured LVEF values (49%–73%). There was a significant underestimation of both LVEDV (Figure 6d) and LVESV (Figure 6e) values by 5D with biases of −4.8 mL and −11.5 mL, respectively. The underestimation of both LVEDV and ESV resulted in a significant LVEF (Figure 6f) bias of 5.6%. The 95% limits of agreement ranged from −28.9 to 19.3 mL for LVEDV, from −32.9 to 9.9 mL for LVESV, and from −3.7% to 14.9% for LVEF. Overall, 5D exhibited high reproducibility when compared to 2D, as evidenced by excellent ICC (and SEM) values of 0.96 (8.9 mL) for LVEDV, 0.82 (7.8 mL) for LVESV, but only fair agreement 0.51 (3.3%) for EF.

**FIGURE 7:**
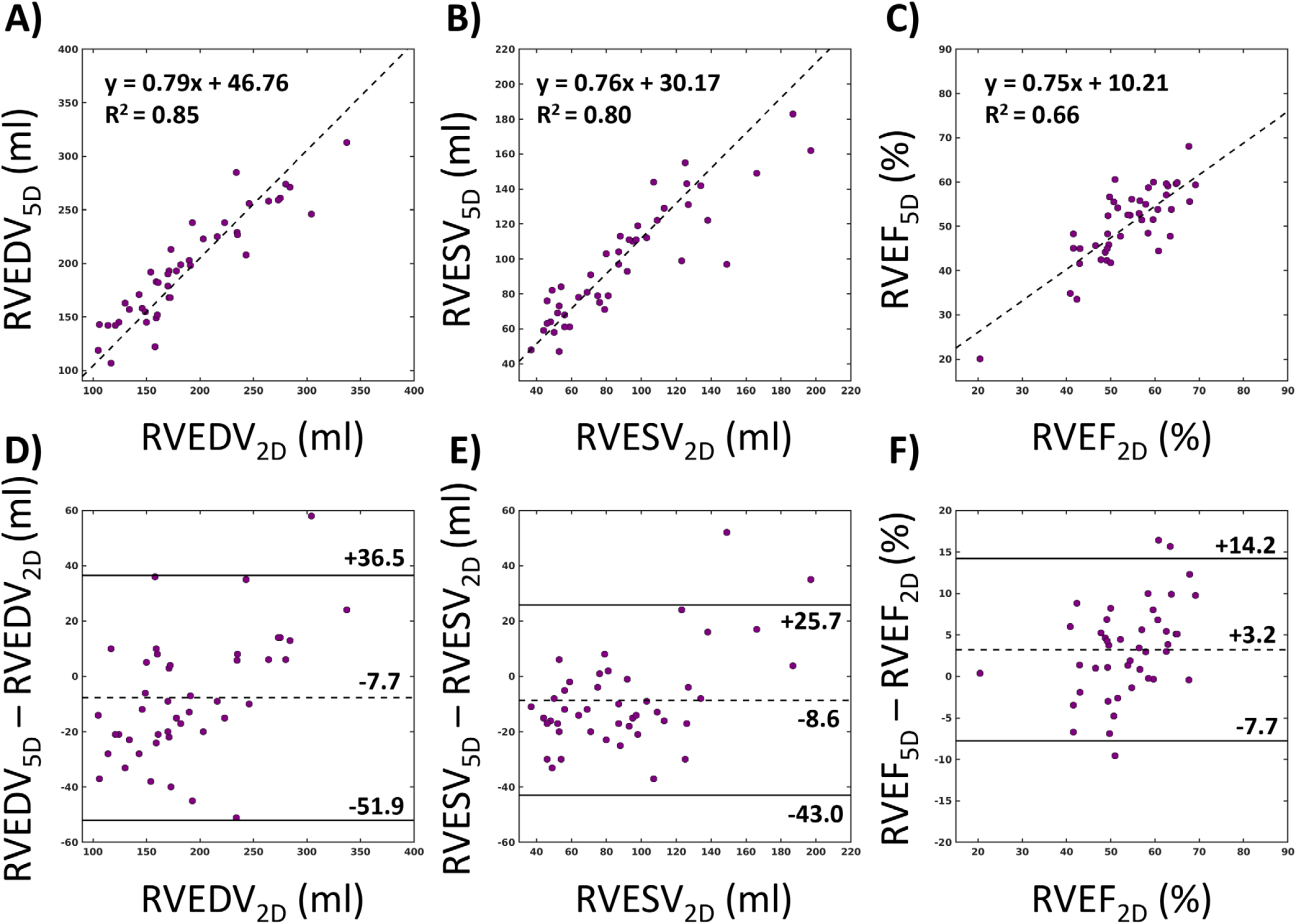
Quantitative evaluation of right ventricular volumetry derived from 2D and 5D imaging data. End‐diastolic volume (A&D), end‐systolic volume (B&E), and ejection fraction (C&F) are assessed by correlation plots (A–C) and Bland–Altman analyses (D–F).

For the right ventricle, very strong correlation (Figure 7) was found between 2D and 5D measurements of RVEDV (*R* ^2^ = 0.85), RVESV (*R* ^2^ = 0.80), and RVEF (*R* ^2^ = 0.66) across the range of measured RVEF values (20%–79%). There was a significant underestimation of both RVEDV (Figure 7d) and RVESV (Figure 7e) values by 5D with biases of −7.7 mL and −8.6 mL, respectively. The underestimation of both LVEDV and ESV resulted in an overestimation of RVEF (Figure 7f) with a bias of 3.2%. The 95% limits of agreement ranged from −51.9 to 36.5 mL for RVEDV, from −43.0 to 25.7 mL for RVESV, and from −7.7%–14.2% for RVEF. Overall, 5D exhibited high reproducibility when compared to 2D, as evidenced by excellent ICC (and SEM) values of 0.90 (15.9 mL) for RVEDV, 0.86 (12.4 mL) for RVESV, and 0.76 (3.9%) for RVEF.

**FIGURE 8:**
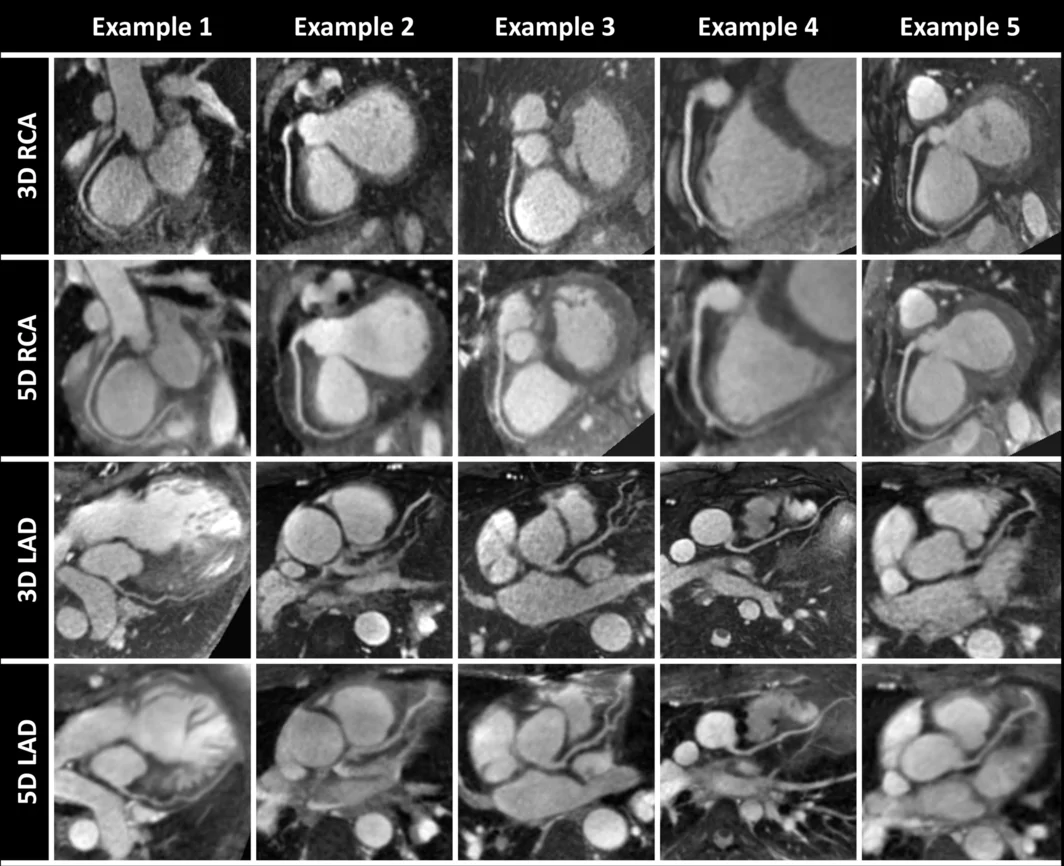
Multi‐planar reformats from 3D and 5D images showing the right (RCA: First and second rows) and left anterior descending (LAD: Third and fourth rows) coronary arteries in five example subjects.

**FIGURE 9:**
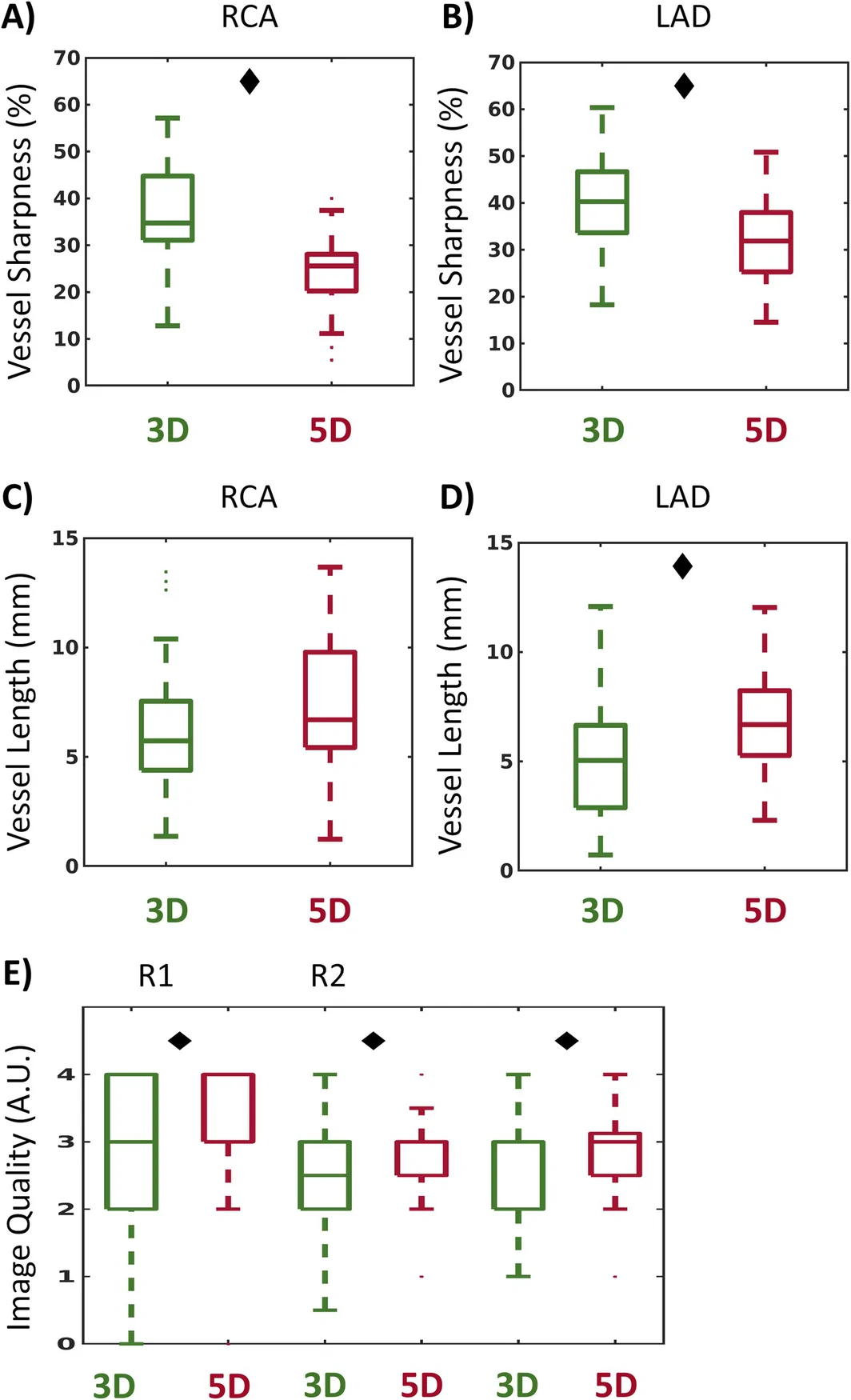
Evaluation of image quality for 3D and 5D whole heart images. Percent vessel sharpness of the RCA (A) and LAD (B), total visible length of the RCA (C) and LAD (D), and Likert‐scale grading by three reviewers (E) are represented by boxplots giving the total range, with the median indicated by the central mark, and the 25th and 75th percentiles by the bottom and top box edges, respectively. Black diamonds denote significant differences between the two imaging methods.

Multiplanar reformatting of 3D and 5D data was successful in all subjects allowing for visualization of the RCA and LAD (Figure 8). Vessel sharpness measured by 3D was significantly higher for both the RCA (Figure 9a: 36.8% ± 10.2%) and LAD (Figure 9b: 40.5% ± 10.5%) when compared to 5D measurements in the RCA (24.3% ± 7.5%) and LAD (31.7% ± 8.7%). The length of the RCA (Figure 9c: 5.4 ± 2.9 mm) measured by 3D was not significantly different than measured by 5D (Figure 9d: 6.6 ± 3.5 mm, *p* = 0.2) but the length of the LAD measured by 3D (5.3 ± 2.9 mm) was significantly shorter than 5D (6.9 ± 2.5).

Qualitative assessment by three reviewers yielded overall higher quality for 5D relative to 3D (Figure 9e). For all three observers, a higher percentage of diagnostically useful images (grade > 2) were found using 5D (R1: 98%, R2: 98%, R3: 98%) relative to 3D (R1: 87%, R2: 82%, R3: 78%). The grades for 5D (R1: 3 [1], R2: 2.5 [0.5], R3: 3 [0.6]) and 3D (R1: 3 [1], R2: 2.5 [1], R3: 2 [1]) were overall significantly different.

## 4 Discussion

Gd contrast enhancement combined with the free‐running FISS sequence resulted in high quality dynamic whole‐heart images of CHD patients. Quantitative comparisons showed that Gd‐enhanced 5D FISS provided results that were not significantly different from those of reference standard 2D CINE and 3D whole‐heart imaging in a significantly shorter scan time. Free‐running whole‐heart FISS therefore presents a highly efficient imaging method with a simplified setup that does not require prospective slice prescription, predefined acquisition windows, ECG gating, breath‐holding, or navigator placement.

Despite the use of Gd contrast for 3D and 5D, 2D imaging still had the highest blood‐myocardium contrast and interface sharpness, but no significant difference was found between 3D and 5D for either of these metrics. Further improvement of 3D and 5D contrast may be possible with the use of tailored slab‐selective excitations but with a likely trade‐off of increased repetition time that could impact the achievable fat signal suppression. Interestingly, neither blood‐myocardium contrast nor blood‐LV interface sharpness exhibited any noticeable trend as a function of time post injection of Gd. This further suggests the flexibility of the 5D approach which may aid in its integration into clinical exams.

Overall, the left and right ventricular borders were well defined in the 5D images and, while significantly less sharp than that of 2D images, enabled subsequent ventricular volume measurements. While strong correlations were found between 2D and 5D ventricular volume and ejection fraction measurements, significant biases remain warranting further study to understand these differences which may come from the highly undersampled 5D data and subsequent compressed sensing reconstruction or inherent differences between 2D breath held and free‐breathing 5D imaging. Further study may include consistency measurements and comparisons to other approaches such as flow‐based measurements of stroke volumes.

Excellent fat signal suppression was achieved using FISS as demonstrated by the blood‐fat contrast being significantly higher for 5D than 2D (where signal from fat does not need to be suppressed) and being not significantly different to the established fat suppressed 3D approach. The resulting 5D images consequently enabled assessment of the origins of both the RCA and LAD. While the overall vessel sharpness was significantly lower for 5D than 3D, it was possible to visualize a longer segment of some vessels using 5D, likely highlighting the trade‐off between signal‐to‐noise ratio and acquired spatial resolution, which was generally poorer for 5D (1.25 mm [3]) than 3D (1.14–1.25 mm [3]). Still, all three reviewers gave 98% of 5D images a grade of 2 (moderate blurring diagnostic value) or higher relative to 87%, 82%, and 78% for 3D indicating that 5D imaging is at least as sufficient for clinical decision‐making as the current 3D approach. These promising results warrant further study on the maximum achievable spatial resolution for the 5D technique and comparison to recent advances in sub‐millimeter 3D whole‐heart imaging [32]. While achieving such higher spatial resolution will require higher scan times, the current 5D protocol could be approximately doubled and still take less time than having to acquire both 2D and 3D sequences.

Together, the qualitative and quantitative comparisons made between 2D, 3D, and 5D thoroughly evaluated the performance of 5D FISS in assessing function and anatomy in a single scan. Among the multiple comparisons, ventricular volume analysis provides an important benchmark that, while still needing improvement, moves us towards a more simplified and efficient acquisition of clinically important markers. Similarly, comparisons of image quality including vessel sharpness and Likert‐scale grading demonstrate the ability to assess CHD anatomy using 5D FISS warranting further study to assess its overall diagnostic accuracy and precision for important anatomical measurements such as aortic diameters [33] and coronary anomalies [5].

## 5 Limitations

In the current study, the proposed 5D approach was compared with reference standard 2D and 3D and demonstrated good performance for conventional clinical markers of interest. However, 5D imaging provides unique information that needs to be further evaluated to define the potential added value of the 5D approach. This includes the ability to visualize complex anatomical structures in 3D while they are moving, which may be important for understanding the relationship between abnormal vessels in CHD. Additionally, the respiratory dimension of the 5D data was unexplored in this study but has previously been shown to provide a measure of changes in hemodynamics [34, 35, 36] in CHD patients and may warrant further exploration of its impact on cardiac function [37].

Despite the simplified and efficient data acquisition, the offline reconstruction times (> 8 h) for 5D imaging remain a significant barrier for clinical translation. However, ongoing developments in deep‐learning based reconstruction may bridge this gap and provide efficient reconstruction of 5D data at the scanner [38]. Similarly, the wealth of data generated by 5D imaging creates a challenge for analyzing the volumetric data. In the current study, short axis reformats were created to match 2D data and therefore leverage existing commercial software for ventricular volumetry. Dedicated software for 3D CINE, while not yet available, is needed to further understand the full clinical utility of 5D imaging.

Previous studies have clearly demonstrated the value of ferumoxytol enhanced 4D and 5D imaging for CHD patients [5, 9, 11, 12]. In the current study we show similar advantages in terms of scan simplification and robustness across heart rate variability by enabling the retrospective selection of arbitrary scan planes and time points in the cardiac cycle. However, Gd is frequently used as a contrast agent in clinical CHD protocols [2, 39] whereas ferumoxytol is used off‐label potentially limiting dissemination of 4D and 5D techniques. Furthermore, the use of FISS in the current study provided native fat suppression which may improve delineation of coronary arteries and reduce artifact from fat. It would therefore be logical to compare Gd‐enhanced 5D FISS to a ferumoxytol‐enhanced 5D approach. This may be challenging to do in the same patients but could be performed with age and sex matched subjects in order to understand the diagnostic advantages of the 5D FISS approach in the context of specific CHD subtypes. Nevertheless, there remains an effort to limit the use of contrast agents due to their cost and reported side effects and therefore greater dissemination of the 5D method would significantly benefit from further advances using native contrast including advances in preparation modules [26] and methods for fat suppression [40, 41, 42].

## 6 Conclusion

Gd‐enhanced free‐running 5D FISS provided dynamic whole heart images that may be used to assess cardiac function and morphology. 5D FISS was acquired in a fixed and significantly shorter acquisition time than reference standard 2D CINE and 3D imaging and multiple quantitative measurements did not differ significantly between techniques.

## Funding

Matthias Stuber is the principal investigator on the Swiss National Science Foundation Grants 320030_173129 and 201292 that funded part of this research. Christopher W. Roy is the principal investigator on Swiss National Science Foundation Grant PZ00P3_202140 that funded part of this research. Jerome Yerly is the principal investigator on Swiss National Science Foundation Grant 310030_215604. Tobias Rutz is the principal investigator on Swiss National Science Foundation Grant 10003134.

## Supporting information


**Table S1:** Patient demographics and characteristics.

## References

[1] A. L. Dorfman , T. Geva , M. M. Samyn , et al., “SCMR Expert Consensus Statement for Cardiovascular Magnetic Resonance of Acquired and Non‐Structural Pediatric Heart Disease,” Journal of Cardiovascular Magnetic Resonance 24 (2022): 44. 35864534 10.1186/s12968-022-00873-1 PMC9302232

[2] S. Fratz , T. Chung , G. F. Greil , et al., “Guidelines and Protocols for Cardiovascular Magnetic Resonance in Children and Adults With Congenital Heart Disease: SCMR Expert Consensus Group on Congenital Heart Disease,” Journal of Cardiovascular Magnetic Resonance 15 (2013): 51. 23763839 10.1186/1532-429X-15-51 PMC3686659

[3] K. C. Odegard , J. A. DiNardo , B. Tsai‐Goodman , A. J. Powell , T. Geva , and P. C. Laussen , “Anaesthesia Considerations for Cardiac MRI in Infants and Small Children,” Paediatric Anaesthesia 14 (2004): 471–476. 15153209 10.1111/j.1460-9592.2004.01221.x

[4] L. Di Sopra , D. Piccini , S. Coppo , M. Stuber , and J. Yerly , “An Automated Approach to Fully Self‐Gated Free‐Running Cardiac and Respiratory Motion‐Resolved 5D Whole‐Heart MRI,” Magnetic Resonance in Medicine 82 (2019): 2118–2132. 31321816 10.1002/mrm.27898

[5] C. W. Roy , K. K. Whitehead , D. Piccini , et al., “Free‐Running Cardiac and Respiratory Motion‐Resolved 5D Whole‐Heart Coronary Cardiovascular Magnetic Resonance Angiography in Pediatric Cardiac Patients Using Ferumoxytol,” Journal of Cardiovascular Magnetic Resonance 24 (2022): 39. 35754040 10.1186/s12968-022-00871-3 PMC9235103

[6] S. A. Si‐Mohamed , L. Romanin , M. Falcao , et al., “Free‐Running 5D Whole‐Heart MRI With Ferumoxytol Enhancement to Evaluate Cardiac Function in Congenital Heart Disease,” International Society for Magnetic Resonance in Medicine 31 (2023): 0556.

[7] R. Nezafat , D. Herzka , C. Stehning , D. C. Peters , K. Nehrke , and W. J. Manning , “Inflow Quantification in Three‐Dimensional Cardiovascular MR Imaging,” Journal of Magnetic Resonance Imaging 28 (2008): 1273–1279. 18972337 10.1002/jmri.21493

[8] Y. Amano , F. Yanagisawa , M. Tachi , et al., “Three‐Dimensional Cardiac MR Imaging: Related Techniques and Clinical Applications,” Magnetic Resonance in Medical Sciences 16 (2017): 183–189. 28202854 10.2463/mrms.rev.2016-0116 PMC5600024

[9] Z. Zhou , F. Han , S. Rapacchi , et al., “Accelerated Ferumoxytol‐Enhanced 4D Multiphase, Steady‐State Imaging With Contrast Enhancement (MUSIC) Cardiovascular MRI: Validation in Pediatric Congenital Heart Disease,” NMR in Biomedicine 30 (2017): 30. 10.1002/nbm.3663 PMC5298926 27862507

[10] J. P. Finn , K.‐L. L. Nguyen , F. Han , et al., “Cardiovascular MRI With Ferumoxytol,” Clinical Radiology 71 (2016): 796–806. 27221526 10.1016/j.crad.2016.03.020 PMC10202101

[11] K.‐L. Nguyen , R. M. Ghosh , L. M. Griffin , et al., “Four‐Dimensional Multiphase Steady‐State MRI With Ferumoxytol Enhancement: Early Multicenter Feasibility in Pediatric Congenital Heart Disease,” Radiology 300 (2021): 162–173. 33876971 10.1148/radiol.2021203696 PMC8248952

[12] J. Y. Cheng , K. Hanneman , T. Zhang , et al., “Comprehensive Motion‐Compensated Highly Accelerated 4D Flow MRI With Ferumoxytol Enhancement for Pediatric Congenital Heart Disease,” Journal of Magnetic Resonance Imaging 43 (2016): 1355–1368. 26646061 10.1002/jmri.25106 PMC4865413

[13] G. M. R. Bongiolatti , M. Prša , K. Pierzchała , et al., “Dynamic Whole‐Heart MRI of Congenital Heart Disease Patients in the Presence of Turbulent Flow,” Journal of Cardiovascular Magnetic Resonance (2026): 102702. 41621594 10.1016/j.jocmr.2026.102702

[14] K.‐L. Nguyen , T. Yoshida , N. Kathuria‐Prakash , et al., “Multicenter Safety and Practice for Off‐Label Diagnostic Use of Ferumoxytol in MRI,” Radiology 293 (2019): 554–564. 31638489 10.1148/radiol.2019190477 PMC6884068

[15] M. A. Fogel , S. Anwar , T. Johnson , et al., “Society for Cardiovascular Magnetic Resonance/European Society of Cardiovascular Imaging/American Society of Echocardiography/Society for Pediatric Radiology/North American Society for Cardiovascular Imaging Guidelines for the Use of Cardiovascular Magnetic Resonance in Pediatric Congenital and Acquired Heart Disease: Endorsed by The American Heart Association,” Journal of Cardiovascular Magnetic Resonance 24 (2022): 37. 35725473 10.1186/s12968-022-00843-7 PMC9210755

[16] I. Koktzoglou and R. R. Edelman , “Radial Fast Interrupted Steady‐State (FISS) Magnetic Resonance Imaging,” Magnetic Resonance in Medicine 79 (2018): 2077–2086. 28856788 10.1002/mrm.26881 PMC5811387

[17] J. A. M. Bastiaansen , D. Piccini , C. W. Roy , et al., “Natively Fat‐Suppressed 5D Whole‐Heart MRI With a Radial Free‐Running Fast‐Interrupted Steady‐State (FISS) Sequence at 1.5T and 3T,” Magnetic Resonance in Medicine 83 (2020): 45–55. 31452244 10.1002/mrm.27942 PMC6778714

[18] Y. Safarkhanlo , J. Yerly , M. B. L. Falcão , et al., “Comparison Between Fast‐Interrupted Steady‐State (FISS) and Rapid Water‐Excitation Pulses for Fat Signal Suppression in Free‐Running Whole‐Heart MRI at 1.5 T,” Magma 38 (2025): 1023–1038. 40549260 10.1007/s10334-025-01273-z PMC12638339

[19] D. Piccini , A. Littmann , S. Nielles‐Vallespin , and M. O. Zenge , “Respiratory Self‐Navigation for Whole‐Heart Bright‐Blood Coronary MRI: Methods for Robust Isolation and Automatic Segmentation of the Blood Pool,” Magnetic Resonance in Medicine 68 (2012): 571–579. 22213169 10.1002/mrm.23247

[20] D. Piccini , P. Monney , C. Sierro , et al., “Respiratory Self‐Navigated Postcontrast Whole‐Heart Coronary MR Angiography: Initial Experience in Patients,” Radiology 270 (2014): 378–386. 24471387 10.1148/radiol.13132045

[21] M. H. Albrecht , A. Varga‐Szemes , U. J. Schoepf , et al., “Coronary Artery Assessment Using Self‐Navigated Free‐Breathing Radial Whole‐Heart Magnetic Resonance Angiography in Patients With Congenital Heart Disease,” European Radiology 28 (2018): 1267–1275. 28887662 10.1007/s00330-017-5035-1

[22] P. Monney , D. Piccini , T. Rutz , et al., “Single Centre Experience of the Application of Self Navigated 3D Whole Heart Cardiovascular Magnetic Resonance for the Assessment of Cardiac Anatomy in Congenital Heart Disease,” Journal of Cardiovascular Magnetic Resonance 17 (2015): 55. 26156377 10.1186/s12968-015-0156-7 PMC4496886

[23] M. B. L. Falcão , A. L. C. Mackowiak , G. M. C. Rossi , et al., “Combined Free‐Running Four‐Dimensional Anatomical and Flow Magnetic Resonance Imaging With Native Contrast Using Synchronization of Neighboring Acquisitions by Physiological Signals,” Journal of Cardiovascular Magnetic Resonance 26 (2024): 101006. 38309581 10.1016/j.jocmr.2024.101006 PMC11211232

[24] C. W. Roy , B. Milani , J. Yerly , et al., “Intra‐Bin Correction and Inter‐Bin Compensation of Respiratory Motion in Free‐Running Five‐Dimensional Whole‐Heart Magnetic Resonance Imaging,” Journal of Cardiovascular Magnetic Resonance 26 (2024): 101037. 38499269 10.1016/j.jocmr.2024.101037 PMC10987330

[25] J. Yerly , C. W. Roy , B. Milani , K. Eyre , M. J. Raifee , and M. Stuber , “High on Sparsity: Interbin Compensation of Cardiac Motion for Improved Assessment of Left‐Ventricular Function Using 5D Whole‐Heart MRI,” Magnetic Resonance in Medicine 93 (2025): 975–992. 39385350 10.1002/mrm.30323 PMC11680726

[26] A. C. Ogier , I. Montón Quesada , X. Sieber , et al., “Free‐Running 5D Whole‐Heart MRI for Isotropic Cardiac Function Measurements at 3T Without Contrast Agents,” Magnetic Resonance in Medicine 93 (2025): 2386–2400. 40035180 10.1002/mrm.30469

[27] C. W. Roy , J. Heerfordt , D. Piccini , et al., “Motion Compensated Whole‐Heart Coronary Magnetic Resonance Angiography Using Focused Navigation (fNAV),” (2020). 10.1186/s12968-021-00717-4 PMC8006382 33775246

[28] R. Ahmad , Y. Ding , and O. P. Simonetti , “Edge Sharpness Assessment by Parametric Modeling: Application to Magnetic Resonance Imaging,” Concepts in Magnetic Resonance Part A 44 (2015): 138–149. 10.1002/cmr.a.21339 PMC4706083 26755895

[29] A. Etienne , R. M. Botnar , A. M. C. van Muiswinkel , P. Boesiger , W. J. Manning , and M. Stuber , “Soap‐Bubble Visualization and Quantitative Analysis of 3D Coronary Magnetic Resonance Angiograms,” Magnetic Resonance in Medicine 48 (2002): 658–666. 12353283 10.1002/mrm.10253

[30] P. Schober , C. Boer , and L. A. Schwarte , “Correlation Coefficients: Appropriate Use and Interpretation,” Anesthesia and Analgesia 126 (2018): 1763–1768. 29481436 10.1213/ANE.0000000000002864

[31] D. V. Cicchetti , “Guidelines, Criteria, and Rules of Thumb for Evaluating Normed and Standardized Assessment Instruments in Psychology,” Psychological Assessment 6 (1994): 284–290.

[32] A. Bustin , I. Rashid , G. Cruz , et al., “3D Whole‐Heart Isotropic Sub‐Millimeter Resolution Coronary Magnetic Resonance Angiography With Non‐Rigid Motion‐Compensated PROST,” Journal of Cardiovascular Magnetic Resonance 22 (2020): 24. 32299445 10.1186/s12968-020-00611-5 PMC7161114

[33] E. Tenisch and T. Rutz , “The Unmet Needs for Aortic Diameter Determination in Patients With Aortopathies,” International Journal of Cardiology 393 (2023): 131299. 37657669 10.1016/j.ijcard.2023.131299

[34] L. E. Ma , J. Yerly , D. Piccini , et al., “5D Flow MRI: A Fully Self‐Gated, Free‐Running Framework for Cardiac and Respiratory Motion‐Resolved 3D Hemodynamics,” Radiology: Cardiothoracic Imaging 2 (2020): e200219. 33385164 10.1148/ryct.2020200219 PMC7755133

[35] E. K. Weiss , J. Baraboo , M. B. L. Falcão , et al., “Respiratory‐Resolved Five‐Dimensional Flow Cardiovascular Magnetic Resonance : In‐Vivo Validation and Respiratory‐Dependent Flow Changes in Healthy Volunteers and Patients With Congenital Heart Disease,” Journal of Cardiovascular Magnetic Resonance 26 (2024): 101077. 39098573 10.1016/j.jocmr.2024.101077 PMC11417305

[36] R. Bastkowski , R. Bindermann , K. Brockmeier , K. Weiss , D. Maintz , and D. Giese , “Respiration Dependency of Caval Blood Flow in Patients With Fontan Circulation: Quantification Using 5D Flow MRI,” Radiology: Cardiothoracic Imaging 1 (2019): e190005. 33778515 10.1148/ryct.2019190005 PMC7977808

[37] J. Liu , Y. Wang , Z. Wen , et al., “Extending Cardiac Functional Assessment With Respiratory‐Resolved 3D Cine MRI,” Scientific Reports 9 (2019): 11563. 31399608 10.1038/s41598-019-47869-z PMC6689015

[38] K. Borsos , M. Stuber , M. Prsa , et al., Efficient Deep‐Learning‐Based Reconstruction of Ferumoxytol‐Enhanced Whole‐Heart 5D Cardiac MRI (International Society for Magnetic Resonance in Medicine, 2024).

[39] H. Baumgartner , M. Chessa , G.‐P. Diller , et al., “2020 ESC Guidelines for the Management of Adult Congenital Heart Disease,” European Heart Journal 42 (2021): 563–645. 32860028 10.1093/eurheartj/ehaa554

[40] N. Masala , J. A. M. Bastiaansen , J. Yerly , et al., “Free‐Running 5D Coronary MR Angiography at 1.5T Using LIBRE Water Excitation Pulses,” Magnetic Resonance in Medicine 84 (2020): 1470–1485. 32144824 10.1002/mrm.28221

[41] X. Sieber , L. Romanin , J. A. M. Bastiaansen , et al., “A Flexible Framework for the Design and Optimization of Water‐Excitation RF Pulses Using B‐Spline Interpolation,” Magnetic Resonance in Medicine 93 (2024): 1896–1910. 39652471 10.1002/mrm.30390

[42] A. L. C. Mackowiak , D. Piccini , and J. A. M. Bastiaansen , “Fat‐Free Noncontrast Whole‐Heart Cardiovascular Magnetic Resonance Imaging With Fast and Power‐Optimized Off‐Resonant Water‐Excitation Pulses,” Journal of Cardiovascular Magnetic Resonance 26 (2024): 101096. 39278414 10.1016/j.jocmr.2024.101096 PMC11616052

