# Supplementary material for: Performance of Free‐Running Cardiac and Respiratory Motion‐Resolved Whole‐Heart 5D MRI in Congenital Heart Disease Patients Using a Gadolinium Enhanced Fast‐Interrupted Steady‐State Sequence
Source: J Magn Reson Imaging. 2026 Jun 24;64(4):996–1008. doi: 10.1002/jmri.70402 (PMC13578529; doi:10.1002/jmri.70402)
Supplement: Supplementary file 1 [file JMRI-64-996-s001.docx]

# Supplementary Material

**Supporting Table 1. Patient demographics and characteristics**

| **#** | **Age** (years) | **Sex** (M/F) | **Heart Rate**  (bpm) | **Diagnosis** | **Characteristics** (surgical procedures, residual defects) |
| --- | --- | --- | --- | --- | --- |
| **1** | 27.1 | M | 87 | Ross operation | Status post-aortic valve replacement. Status post Ross operation. Status post Tirone David operation, aortic valve repair, autograft Contegra replacement and obliteration of RV aneurysm. Residual aortic insufficiency and supravalvular gradient. |
| **2** | 27.0 | M | 61 | Tetralogy of Fallot | Status post complete correction and Contegra implantation. Residual pulmonary insufficiency and pulmonary stenosis. |
| **3** | 26.5 | M | 57 | Tetralogy of Fallot | Status post correction, pulmonary insufficiency. |
| **4** | 62.9 | F | 65 | Pulmonary stenosis | Aortic stenosis. |
| **5** | 32.5 | F | 64 | Transposition of the great arteries | Status post Senning. |
| **6** | 63.2 | M | 68 | Bicuspid aortic valve | Status post aortic dilatation |
| **7** | 56.3 | F | 71 | Aortic coarctation (Turner Syndrome) | Status-post coarctation resection. |
| **8** | 46.5 | M | 54 | Aortic coarctation | Status-post coarctation resection. Status-post percutaneous balloon angioplasty. Post operative subclavian artery lesion. |
| **9** | 26.2 | M | 60 | Pulmonary stenosis | Status-post percutaneous dilation and surgical commissurotomy. |
| **10** | 47.7 | F | 60 | Tetralogy of Fallot | Status-post pulmonary valve replacement with a right-to-left homograft |
| **11** | 74.7 | F | 80 | Bicuspid aortic valve | Status-post replacement of the ascending aorta and reduction of non-coronary sinuses. Double aortic arch. |
| **12** | 52.0 | M | 73 | Tetralogy of Fallot | Status-post correction and Hancock bio prosthesis |
| **13** | 22.3 | F | 75 | Aortic coarctation, VSD | Status post stenting and aortic dilation. |
| **14** | 22.4 | F | 68 | Aortic coarctation (Turner Syndrome) |  |
| **15** | 27.8 | M | 63 | VSD | Status-post VSD closure |
| **16** | 45.7 | M | 74 | Bicuspid aortic valve |  |
| **17** | 39.3 | F | 66 | Ebstein disease | Status-post Hetzer valvuloplasty. |
| **18** | 36.7 | F | 63 | Pulmonary atresia without VSD | Status-post pulmonary commissurotomy. Status-post repair by transannular patch. Moderate homograft stenosis. |
| **19** | 22.2 | M | 81 | ASD | Status-post correction. Left superior vena cava draining in coronary sinus. |
| **20** | 28.2 | F | 72 | Pulmonary stenosis | Status-post balloon angioplasty. |
| **21** | 34.5 | M | 49 | Transposition of the great arteries | Status-post arterial switch. |
| **22** | 57.8 | F | 66 | D-transposition of the great arteries | Status-post Blalock Halon and interarticular communication creation. Status-post arterial switch (Mustard). |
| **23** | 55.6 | F | 74 | Patent arterial duct | Status-post closure and tube aortic reparation. Tube stenosis. |
| **24** | 27.9 | F | 77 | Pulmonary stenosis |  |
| **25** | 23.7 | F | 56 | Loeys-Dietz syndrome |  |
| **26** | 64.3 | M | 58 | Bicuspid aortic valve | Status-post Tirone David operation. |
| **27** | 43.4 | M | 76 | Marfan syndrome |  |
| **28** | 25.3 | F | 61 | Tetralogy of Fallot | Status-post repair by transannular patch and Hancock valved conduit. |
| **29** | 23.2 | M | 47 | D-transposition of the great arteries, VSD | Status-post arterial switch and VSD closure. |
| **30** | 39.9 | M | 73 | Bicuspid aortic valve |  |
| **31** | 21.7 | F | 74 | Bicuspid aortic valve, aortic coarctation | Status-post percutaneous dilation. |
| **32** | 30.9 | F | 60 | Pulmonary hypertension | Status-post ASD closure |
| **33** | 33.0 | F | 54 | Tetralogy of Fallot | Status-post correction with homograft. Persistent VD dilation. |
| **34** | 49.3 | M | 55 | Ross procedure | Status-post Ross operation and aortoplasty for dilation. |
| **35** | 23.3 | M | 53 | Loeys-Dietz syndrome | Aortic root dilation. |
| **36** | 57.5 | F | 65 | Pulmonary stenosis, ASD, atrial-septal-aneurysm | Status-post balloon angioplasty |
| **37** | 23.1 | M | 61 | Tetralogy of Fallot | Status-post repair by transannular patch and Hancock bio prothesis. |
| **38** | 37.6 | F | 67 | Aortic arch | Status-post arterial ligament resection |
| **39** | 23.0 | M | 54 | Aortic arch, VSD | Status-post aortic arch correction. |
| **40** | 25.5 | M | 76 | Bicuspid aortic valve, aortic coarctation | Status-post aortoplasty. |
| **41** | 26.5 | M | 70 | Ebstein disease | Status-post tricuspid valve replacement |
| **42** | 52.6 | F | 65 | Pulmonary hypertension | Communication between LPA and descending Aorta |
| **43** | 23.3 | M | 65 | Pulmonary hypertension |  |
| **44** | 22.8 | F | 68 | Patent arterial duct |  |
| **45** | 40.1 | M | 74 | Loeys-Dietz syndrome |  |

*VSD*: ventricular septal defect. *ASD*: atrial septal defect.
